# Change in Activity of Palliative Care Services during the Covid-19 Pandemic: A Multinational Survey (CovPall)

**DOI:** 10.1089/jpm.2021.0315

**Published:** 2022-03-01

**Authors:** Katherine E. Sleeman, Rachel L. Cripps, Fliss E.M. Murtagh, Adejoke O. Oluyase, Mevhibe B. Hocaoglu, Matthew Maddocks, Catherine Walshe, Nancy Preston, Lesley Dunleavy, Andy Bradshaw, Sabrina Bajwah, Irene J. Higginson, Lorna K. Fraser

**Affiliations:** ^1^ Cicely Saunders Institute of Palliative Care, Policy and Rehabilitation, King's College London, London, United Kingdom.; ^2^ King's College Hospital NHS Foundation Trust, Denmark Hill, United Kingdom.; ^3^ Wolfson Palliative Care Research Centre, Hull York Medical School, University of Hull, Hull, United Kingdom.; ^4^ International Observatory on End of Life Care, Division of Health Research, Lancaster University, Lancaster, United Kingdom.; ^5^ Health Sciences, University of York, York, United Kingdom.

**Keywords:** Covid-19, end-of-life care, hospices, palliative care, pandemics, severe acute respiratory syndrome coronavirus 2

## Abstract

**Objectives:**

To identify factors associated with palliative care services being busier during Covid-19.

**Methods:**

Cross-sectional online survey of UK palliative care services (April to July 2020) (CovPall). Ethical approval was received from King's College London Research Ethics committee (LRS-19/20-18541). The primary outcome was change in busyness (five-point ordinal scale). Ordinal logistic regression investigated factors associated with the primary outcome.

**Results:**

Of 277 responses, 71 (26%) reported being a lot more busy, 62 (22%) slightly more, 53 (19%) about the same, 50 (18%) slightly less, and 28 (10%) much less busy. Increased business was associated with homecare services (odds ratio [OR] 1.93, 95% confidence interval [CI] 1.15–3.25), nursing care at home (OR 3.24, 95% CI 1.70–6.19), publicly managed services (OR 2.20, 95% CI 1.11–4.34), Covid-19 cases (OR 1.01, 95% CI 1.00–1.01), and staff shortages (OR 2.71, 95% CI 1.64–4.48).

**Conclusion:**

Services providing community care, and publicly managed services, may have been better able to respond to escalating needs during Covid-19. This has potential implications for both service delivery and funding models.

## Introduction

The Covid-19 pandemic gave rise to a rapid increase in the level of need for palliative care.^1–3^ In parallel, there were changes in patient and family priorities, with people who might otherwise have been admitted to a hospice choosing to be cared for at home due to fear of infection and visiting restrictions.^4,5^ In response, many hospice and palliative care services rapidly innovated, reconfiguring services, increasing community outreach, and adopting new technology for communication with patients, families, and professionals.^6^

During the first months of the pandemic, some hospice and palliative care services reported being more busy, while others reported being less busy than before.^7^ We know little about which services experienced increased activity levels, and which experienced reduced activity levels. Understanding whether services that reported becoming more (or less) busy share certain characteristics could help identify strategies and/or structures to maximize the contribution of hospice and palliative care services to the wider health and social care system, particularly during current and future pandemics. The aim was to identify factors associated with palliative care and hospice services being busier during the Covid-19 pandemic.

## Methods

### Study design and participants

CovPall is a multicenter multinational observational study of specialist palliative care during the Covid-19 pandemic. The first component of CovPall was an online survey of palliative care services (opened on April 23rd, 2020, and closed on July 31st, 2020); detailed methods, including the full survey, have been reported previously.^7^ Ethical approval was received from King's College London Research Ethics committee (LRS-19/20-18541). The survey is reported according to the MORECARE^8^ statement.

### Procedures and questionnaire

Survey procedures have been previously described.^7^ In brief, services were contacted through palliative care and hospice organizations and provided with a link to the brief (∼30 minutes) online participant information sheet and survey. Data were anonymized before analysis.

### Inclusion criteria

For this study, we limited analysis to responses from the four nations of the United Kingdom (England, Scotland, Wales, and Northern Ireland) to reduce heterogeneity and enable us to draw more meaningful conclusions. In the United Kingdom, hospice and palliative care services work across community (home and care home), inpatient hospice unit, and hospital settings (inpatient advisory teams), and provide care for adults and children. Management of services varies, with around 30% of hospice funding from public sources.^9,10^

### Analysis

For the analysis, the primary outcome was change in busyness (“Would you say overall you are more busy or less busy than before the Covid-19 Pandemic?”), measured using a five-point ordinal scale (1 = much less busy, 2 = a little less busy, 3 = about the same, 4 = a little more busy, and 5 = a lot more busy).^7^ We used descriptive statistics to explore the relationship between change in busyness and explanatory variables.

Explanatory variables included those related to (1) service organization: funding model (public, charitable, and other) and type of service provided (inpatient hospice unit, hospital advisory team, specialist palliative home care service, and hands-on nursing care in the community); (2) clinical factors: number of confirmed (by test) cases of Covid-19 (continuous variable), number of suspected cases of Covid-19 (continuous variable), personal protective equipment (yes/no), medication shortages (yes/no), and staff shortages (yes/no); and (3) geography: Scotland, Northern Ireland, Wales, and the nine regions of England. The population size of Scotland, Northern Ireland, and Wales approximates to that of the regions of England.

Unadjusted ordinal regression was used to examine the relationship between explanatory variables and the outcome (a higher level of busyness). For the multivariable model, explanatory variables were selected according to *a priori* hypotheses (that busyness would vary in relation to setting, management type, and number of Covid-19 cases) and significance in unadjusted analyses (*p* < 0.1), after checking that the assumptions for ordinal logistic regression (absence of multicollinearity and presence of proportional odds) had been met.^11^ Although this was a cross-sectional survey, our *a priori* hypotheses paid attention to the likely temporal sequence between potential cause and effect.

We were interested in factors that might lead to being more busy, rather than those that were more likely to be a consequence of being busy. The decision of whether variables were considered factors that led to being more busy or a consequence of being more busy was made by the CovPall Study Steering Group after discussion. A sensitivity analysis was performed in which services that exclusively provided children's services were excluded, as Covid-19 is likely to have affected children's services differently to adults' services.^12^ Analysis was performed in Stata v16 (StataCorp).^13^

## Results

There were 277 responses from clinical leads (medical director/lead medical clinician, nurse director/lead nurse clinician, or other) of UK palliative care services: 33 from Scotland, 4 from Northern Ireland, 15 from Wales, and 225 from the nine regions of England. Many services provided care in more than one setting; 168 (61%) provided inpatient hospice services, 135 (49%) provided hospital advisory teams, 160 (58%) provided home care services, and 92 (33%) provided hands-on care in the community. Sixteen services (6%) provided children-only services. One hundred forty-three services (52%) were charitably managed and 103 (37%) were publicly managed. Table 1 describes busyness according to service level, clinical and geographical variables.

**Table 1.** Characteristics of Palliative Care Services by Busyness, and Unadjusted and Multivariable Ordinal Logistic Regression to Identify Factors Associated with Hospice and Palliative Care Services Reporting Being More Busy

	Descriptive statistics: busyness						Unadjusted analysis					Multivariable analysis (*N* = 241)			
	Much less busy (*N* = 28)	Slightly less busy (*N* = 50)	About the same (*N* = 53)	Slightly more busy (*N* = 62)	A lot more busy (*N* = 71)	Missing (*N* = 13)	Total Sample (*N* = 277)	OR	CI lower	CI upper	*p*	OR	CI lower	CI upper	*p*
Setting, *n* (%)^a^															
Inpatient hospice/palliative care unit—yes (ref no)	18 (11)	32 (19)	33 (20)	36 (21)	39 (23)	10 (6)	168	0.76	0.49	1.18	0.23				
Hospital palliative care advisory team—yes (ref no)	13 (10)	24 (18)	22 (16)	31 (23)	38 (28)	7 (5)	135	1.23	0.80	1.88	0.35				
Specialist palliative home care service—yes (ref no)	10 (6)	28 (18)	27 (17)	43 (27)	43 (27)	9 (6)	160	**1.63**	**1.05**	**2.53**	**0.03**	**1.93**	**1.15**	**3.25**	**0.01**
Providing hands-on nursing care at home/in the community—yes (ref no)	3 (3)	16 (17)	21 (23)	19 (21)	28 (30)	5 (5)	92	1.54	0.98	2.43	0.06	3.24	1.70	6.19	<0.01
Management type, *n* (%)															
Charitable/nonprofit	18 (13)	27 (19)	34 (24)	29 (20)	34 (24)	1 (1)	143	1 (Ref)				1 (Ref)			
Public	7 (7)	21 (20)	15 (15)	27 (26)	32 (31)	1 (1)	103	**1.51**	**0.96**	**2.38**	**0.08**	**2.20**	**1.11**	**4.34**	**0.02**
Other	2 (13)	2 (13)	3 (19)	5 (31)	4 (25)	0 (0)	16	1.30	0.52	3.24	0.57	1.40	0.46	4.25	0.56
Missing	1 (7)	0 (0)	1 (7)	1 (7)	1 (7)	11 (73)	15								
Confirmed number of Covid-19 cases															
Median (IQR)	4 (1–20)	5 (1–41)	5 (1–30)	10 (2–50)	15.5 (7–74)	6	10 (2–50)	**1.01**	**1.00**	**1.01**	**<0.01**	**1.01**	**1.00**	**1.01**	**0.01**
Total	25	48	50	61	66	1	251								
Suspected number of Covid-19 cases															
Median (IQR)	2 (0–8)	4 (0–15)	5.5 (1–13)	6 (2–20)	11.5 (4–27.5)	20	6 (1–20)	**1.01**	**1.00**	**1.01**	**0.04**				
Total	27	47	48	58	64	1	245								
PPE shortages, *n* (%)															
No	19 (15)	25 (19)	20 (16)	34 (26)	31 (24)	0 (0)	129	1 (Ref)							
Yes	9 (7)	25 (19)	33 (26)	26 (20)	36 (28)	0 (0)	129	1.22	0.79	1.88	0.37				
Missing	0 (0)	0 (0)	0 (0)	2 (11)	4 (21)	13 (68)	19								
Medication shortages, *n* (%)															
No	22 (11)	39 (20)	39 (20)	44 (23)	48 (25)	0 (0)	192	1 (Ref)							
Yes	6 (10)	10 (16)	13 (21)	15 (24)	19 (30)	0 (0)	63	1.30	0.78	2.16	0.31				
Missing	0 (0)	1 (5)	1 (5)	3 (14)	4 (18)	13 (59)	22								
Staff shortages, *n* (%)															
No	19 (14)	27 (20)	38 (28)	34 (25)	20 (14)	0 (0)	138	1 (Ref)				1 (Ref)			
Yes	9 (8)	21 (18)	14 (12)	26 (22)	47 (40)	0 (0)	117	**2.50**	**1.59**	**3.93**	**<0.01**	**2.71**	**1.64**	**4.48**	**<0.01**
Missing	0 (0)	2 (9)	1 (5)	2 (9)	4 (18)	13 (59)	22								
Nation/Region, *n* (%)															
South East England	6 (14)	7 (17)	7 (17)	9 (21)	12 (29)	1 (2)	42	1 (Ref)				1 (Ref)			
Scotland	4 (12)	8 (24)	5 (15)	6 (18)	8 (24)	2 (6)	33	0.80	0.34	1.88	0.61	1.72	0.65	4.56	0.27
Wales	0 (0)	6 (40)	1 (7)	6 (40)	2 (13)	0 (0)	15	0.84	0.30	2.34	0.74	1.70	0.51	5.66	0.39
Northern Ireland	0 (0)	0 (0)	2 (50)	2 (50)	0 (0)	0 (0)	4	1.04	0.22	4.97	0.97	1.73	0.30	10.05	0.54
England															
North East	1 (8)	3 (25)	4 (33)	0 (0)	4 (33)	0 (0)	12	0.85	0.26	2.75	0.79	0.89	0.27	2.90	0.85
North West	5 (14)	6 (17)	9 (25)	8 (22)	6 (17)	2 (6)	36	0.72	0.32	1.63	0.44	1.26	0.54	2.96	0.59
Yorkshire and The Humber	3 (12)	3 (12)	4 (15)	9 (35)	5 (19)	2 (8)	26	1.07	0.44	2.62	0.88	2.02	0.75	5.48	0.17
East Midlands	2 (17)	0 (0)	4 (33)	2 (17)	3 (25)	1 (8)	12	1.02	0.31	3.37	0.97	2.39	0.58	9.84	0.23
West Midlands	3 (20)	2 (13)	4 (27)	1 (7)	4 (27)	1 (7)	15	0.68	0.22	2.11	0.51	1.52	0.49	4.72	0.47
East	0 (0)	3 (20)	5 (33)	2 (13)	4 (27)	1 (7)	15	1.13	0.39	3.26	0.82	1.44	0.46	4.52	0.53
London	3 (7)	6 (14)	3 (7)	9 (21)	20 (48)	1 (2)	42	**2.41**	**1.01**	**5.45**	**0.04**	**3.24**	**1.30**	**8.05**	**0.01**
South West	1 (4)	6 (24)	5 (20)	8 (32)	3 (12)	2 (8)	25	0.84	0.35	2.04	0.71	1.43	0.55	3.73	0.46

Bold values are statistically significant results.

Percentages are row percentages.

^a^ Each service could provide care in more than one setting.

CI, confidence interval; IQR, interquartile range; OR, odds ratio; PPE, personal protective equipment.

In unadjusted analyses, being more busy was positively associated with the following: providing a specialist palliative home care service; providing hands-on care in the community; being publicly managed; having more confirmed and suspected cases of Covid-19; reporting staff shortages; and geographical location (Table 1). For the multivariable analysis, we excluded the number of suspected cases of Covid-19 as it correlated closely with confirmed cases. All the included explanatory variables remained statistically significantly associated with being more busy (Table 1). The sensitivity analysis, excluding children-only services, showed similar results (Appendix Table A1).

## Discussion

In this large survey of hospice and palliative care services across the United Kingdom, just under half of services reported being slightly or a lot more busy during the early months of the Covid-19 pandemic, while one in three services reported being slightly or much less busy. Being busier was associated with services that provided hands-on care at home and in the community and home care services, those that were publicly (rather than charitably) managed, those that had experienced more confirmed cases of Covid-19, and those that had experienced staff shortages.

Hospice and palliative care services that provided hands-on and home care services in the community had greater odds of being busier than services that did not provide care in these settings. During the Covid-19 pandemic, there were a shift in patient and family preferences as visiting restrictions and fear of infection meant many people preferred to remain at home rather than go to hospitals or to inpatient hospice units,^4^ and deaths in inpatient hospices fell, while home deaths increased.^5^ With more people choosing to remain at home, services providing care in the community may have been able to respond to these changes in preferences. This is in keeping with findings from a survey of General Practitioners and District Nurses, which found that primary care teams provided both higher volume and higher complexity of community palliative care during the pandemic.^4^

Services that were publicly managed had greater odds of being busier compared to services that were charitably managed. In the United Kingdom, only 30% of hospice funding overall is from public/government sources, with 70% from charitable sources.^9,10^ It is not clear why there should be a difference in busyness according to funding type. A possible explanation is that publicly managed services may be better integrated into the wider health and social care system, and so more able to contribute to a system-wide response. Further investigation is needed.

### Strengths and limitations

This was a large survey, with 277 responses across the United Kingdom. It is estimated that there are 200 hospice services in the United Kingdom^14^; we received a good response rate from these services with 168 (*∼*84%) completing our survey. We measured services' self-reported change in busyness, based on a single question, usually reported by the clinical lead. Busyness is a subjective concept and may be perceived differently by different stakeholders. Correlation with a change in the number of patient consultations or referrals was not possible from the available data. We relied on information collected from clinical service leads, which may have introduced bias. Further research should explore the views of other frontline end-of-life care workers. This survey was not able to capture any change in care provision outside of palliative care and hospice teams as the survey was completed by clinical leads of UK palliative care services.

We also do not have any information about the number of patient consults or referrals to validate how busy services were. Missing data for the variables of interest were low. However, responses were not evenly distributed across the United Kingdom; there were only four responses from Northern Ireland. The survey was carried out between April and July 2020, a period of time during which Covid-19 case numbers across the United Kingdom varied greatly and changed rapidly, for example, London was affected earlier and more severely in the first wave of Covid-19 in the United Kingdom. Adjustment for geographical area will have accounted for some, but not all of the regional variation. Unmeasured confounders such as capacity of services may influence the findings.

The cross-sectional design means that causal relationships cannot be determined. Our *a priori* hypotheses were designed to distinguish between causes of busyness (our interest) and the consequences of it, although this was not always clear. Previous analysis of free text data from CovPall identified increased clinical activity, increased education, and increased use of technology as contributing to busyness of services^15^ However, we cannot rule out other reasons for being busier such as greater administrative burden, or less efficient structures and processes. Therefore, it cannot be inferred that being busier means better patient access; there may be circumstances where busyness detracts from direct patient care rather than contributing to it. Future studies should examine the practical implications of a service being more busy such as quality of care and patient outcomes.

## Conclusion

Being busier was associated with services that provided community care, and those that were publicly managed. This may indicate that service and funding models influence the ability of hospices and palliative care services to respond rapidly to changing needs and priorities. Our study provides a starting point for further research, exploring the ability of hospice and palliative care services to respond rapidly to changing patient preferences and societal needs.

## Appendix

**Appendix Table A1.** Sensitivity Analysis Removing Children-Only Services from the Multivariable Ordinal Logistic Regression

Busyness (Ref—A lot less busy)	Sensitivity analysis(*N* = 227)			
	OR	CI lower	CI upper	*p*
Setting				
Specialist palliative home care service—yes (ref no)	**1.94**	**1.13**	**3.31**	**0.02**
Providing hands on nursing care at home/in the community—yes (ref no)	**3.64**	**1.84**	**7.21**	**<0.01**
Management type				
Charitable/nonprofit	1 (Ref)			
Public	**2.17**	**1.07**	**4.40**	**0.03**
Other	1.33	0.43	4.13	0.62
Confirmed number of Covid-19 cases	**1.01**	**1.00**	**1.01**	**0.02**
Staff shortages—yes (ref no)	**2.53**	**1.51**	**4.24**	**<0.01**
Country/Region				
South East England	1 (Ref)			
Scotland	1.56	0.54	4.48	0.41
Wales	1.46	0.43	4.93	0.55
Northern Ireland	1.43	0.24	8.50	0.69
England				
North East	1.06	0.29	3.92	0.93
North West	1.04	0.43	2.50	0.93
Yorkshire and The Humber	1.59	0.57	4.46	0.38
East Midlands	1.96	0.47	8.28	0.36
West Midlands	1.82	0.55	6.00	0.33
East	1.38	0.42	4.56	0.60
London	**2.80**	**1.11**	**7.09**	**0.03**
South West	1.18	0.44	3.14	0.74

Bold values are statistically significant results.

CI, confidence interval; OR, odds ratio.
